# Cytokine Reduction in the Treatment of Joint Conditions

**DOI:** 10.1155/S0962935194000359

**Published:** 1994

**Authors:** J. D. Sipe, J. Martel-Pelletier, I. G. Otterness, J.-P. Pelletier

**Affiliations:** 1 Department of Biochemistry Boston University School of Medicine Boston MA USA; 2 Rheumatic Disease Unit Notre-Dame Hospital Research Center Montreal Quebec Canada; 3 Department of Immunology and Infectious Diseases Pfizer, Inc. Groton CT USA

## Abstract

The destruction of joints caused by rheumatoid arthritis and osteoarthritis is characterized by an imbalance of enzyme catalysed cartilage breakdown and regeneration. A complex cytokine network perpetuates joint conditions by direct regulation of metalloproteases, by indirect recruitment of cells that secrete degradative enzymes, and by inhibition of reparative processes. The destructive action of cytokines such as interleukin-1, interleukin-6 and tumour necrosis factor-α can be modulated at multiple points associated either with cytokine production or with cytokine action. Potential agents for cytokine reduction include selective anti-cytokine antibodies, anticytokine receptor antibodies, cytokine receptor antagonist proteins, and soluble and chimeric cytokine receptor molecules. Pharmacologic regulation of IL-1 and TNFα remain primary targets for treatment of arthritis, and results of early clinical trials are promising. However, the results of long-term clinical trials will be required to support the value of anti-cytokine therapy in treatment of arthritis.


Invited Review

Mediators of Inflammation 3, 243–256 (1994)

### Cytokine reduction in the treatment of joint conditions

J. D. Sipe,[1,CA] J. Martel-Pelletier,[2] I. G. Otterness[3] and J.-P. Pelletier[2]

[1]Department of Biochemistry, Boston University School of Medicine, Boston, MA, USA; [2]Rheumatic Disease Unit, Notre-Dame Hospital Research Center, Montreal, Quebec, Canada; [3]Department of Immunology and Infectious Diseases, Pfizer, Inc., Groton, CT, USA

[CA]Corresponding Author

**THE destruction of joints caused by rheumatoid arthritis and osteoarthritis is characterized by an imbalance of enzyme catalysed cartilage breakdown and regeneration. A complex cytokine network perpetuates joint conditions by direct regulation of metalloproteases, by indirect recruitment of cells that secrete degradative enzymes, and by inhibition of reparative processes. The destructive action of cytokines such as interleukin-1, interleukin-6 and tumour necrosis factor-α can be modulated at multiple points associated either with cytokine production or with cytokine action. Potential agents for cytokine reduction include selective anti-cytokine antibodies, anti-cytokine receptor antibodies, cytokine receptor antagonist proteins, and soluble and chimeric cytokine receptor molecules. Pharmacologic regulation of IL-1 and TNFα remain primary targets for treatment of arthritis, and results of early clinical trials are promising. However, the results of long-term clinical trials will be required to support the value of anti-cytokine therapy in treatment of arthritis.**

**Key words**: Acute phase proteins, Adhesion molecules, Antiarthritic molecules, Cartilage matrix, Interleukin-1, Interleukin-6, Metalloproteases, Osteoarthritis, Rheumatoid arthritis, Tumour necrosis factor-α

### Introduction

Slow, steady destruction of painful, swollen joints is the hallmark of connective tissue disorders such as rheumatoid arthritis (RA) and osteoarthritis (OA). RA, an autoimmune disease, involves T and B cell infiltration of the synovial lining and extensive proliferation of synovial lining cells, resulting in the formation of pannus and influx of polymorphonucleocytes (PMNs) and monocytes into both the synovium and synovial fluid. There may be considerable bone loss, particularly at the margins of the synovial lining, and joint deformity.[1,2] OA is primarily characterized by loss of articular cartilage. Synovitis may play a role, particularly in painful joints, but bone loss and pannus formation are uncharacteristic. However, cartilage and bone parameters are not normal either in RA or OA. RA and OA are at opposite ends of an inflammation spectrum, in that RA affects multiple joints of the body, involves a large-scale systemic response and is immunologically driven. Cartilage loss in RA proceeds from the invading edge of the pannus. In OA cartilage loss appears to be primarily driven by the cartilage itself. In both cases, joint destruction is characterized by an imbalance of enzyme catalysed cartilage breakdown and regeneration.

While both OA and RA are characterized by increased amounts of metalloproteases in synovium and cartilage, the synovial involvement is far more pronounced in RA. Thus the pathway of joint destruction in RA is thought to overlap the events causing OA, but to be broader in scope and to engage more members of the cytokine network.[3,4] The aetiology of OA is thought to involve precipitating events ranging from crystal deposition, joint neuropathy, sub-chondral sclerosis, mechanical defects, metabolic abnormalities, to mechanical trauma. However, alterations in chondrocyte metabolism are thought to be at the root of OA of various origins.[5]

Various members of the cytokine network have been implicated in joint destruction both by direct regulation of metalloproteases, by indirect recruitment of cells that secrete degradative enzymes, and by inhibition of reparative processes. Among the most well characterized are IL-1, IL-6, TNF and chemokines such as IL-8. The destructive action of cytokines can be modulated at multiple points associated either with cytokine production, including transcription, translation, secretion and degradation; or with cytokine action, including inhibition of receptor binding and signal transduction. In this review, the potential of cytokine modification for treatment of the

© 1994 Rapid Communications of Oxford Ltd destruction of joints accompanying OA and RA will be considered.

### Conditions of joints in osteoarthritis and rheumatoid arthritis

Osteoarthritis (OA) is defined as a complex of interactive degradative and repair processes in cartilage, bone and synovium, with secondary components of inflammation. The aetiopathologic processes involved are complex, and their relative importance continues to be debated.

By currently held concepts, two general pathways lead to OA.[6] The first involves fundamentally defective cartilage with biomaterial properties directly or indirectly leading to OA. Thereby, the cartilage matrix fails under normal loading of the joint. A recently described type II collagen defect well exemplifies this pathway;[7] following biomechanical failure osteoarthritis ensues. The second, and by far most prevalent, concept of the cause of OA is based on the major role that physical forces play in causing damage to normal articular cartilage matrix. First, there is direct injury of the matrix; secondly, chondrocytes embedded in the matrix are injured by the same forces.[8–11] In the course of time, these chondrocytes react to injury by elaborating degradative enzymes and developing inappropriate repair responses.[11,12] Recent research implicates the enzymatic breakdown of cartilage as a key feature of disease progression.[11]

OA is characterized by the increasing degeneration of articular cartilage, a thickening of the subchondral bone, and the formation of marginal osteophytes. Biochemical and histological studies indicate that there is focal loss of extracellular ground substance in the matrix of OA lesions. As the disease progresses, there are ulcerations in the cartilage and, finally, the entire articular surface is lost.[13] In OA, changes not only involve the cartilage, but other joint structures such as the synovial membrane.[14]

Synovial inflammation is responsible for some OA symptoms and is also likely to play an important role in the pathological process by interacting with, and thereby accelerating, catabolism.[11] The order in which the biochemical changes take place during the destructive phase of this disease is not yet clear. However, one of the main biochemical changes in the articular cartilage appears to be related to alterations in the proteoglycan structure. These macromolecules undergo quantitative and qualitative changes.[13,15] There is a progressive depletion of cartilage proteoglycan, which parallels the severity of the disease.[16] At a certain stage, the chondrocytes appear unable to fully compensate for the proteoglycan depletion resulting in a net loss of matrix. The structural changes of the proteoglycan macromolecules include a decrease in hyaluronic acid content, a diminution in the size of proteoglycan aggregates and monomers, and a decrease in the aggregation properties of the monomers.[16–18] The latter changes seem likely to reflect the degradation of the proteoglycan monomer core protein in which cleavage has occurred in several areas, including the hyaluronic acid binding region (HABR).[19] Although the content of type II collagen remains unchanged in OA cartilage, the increased cartilage hydration and the ultrastructural changes of the collagen fibres[20] represent important alterations in the collagen fibre network. The increase in minor collagen types such as type I collagen, particularly in the pericellular area, suggests a change in chondrocyte metabolism.[21] These changes in the proteoglycan content of the matrix, together with the damaged collagen structure, lead to a functional deterioration of the cartilage, making it less resistant to compression and other mechanical stress, which lead to the appearance of progressive cartilage lesions.

In OA, mechanical factors and enzymatic pathways are both involved in cartilage matrix degradation.[11] The enzymatic process appears related not to a unique system, but rather to a cascade of events.[11] In contrast to rheumatoid arthritis (RA), where the synovium is probably the most important source of degradative enzymes, chondrocytes seem to be the most important enzymatic source responsible for OA cartilage matrix metabolism. Currently, the enzyme families that have been identified as playing a significant role in OA pathophysiology are the metalloproteases, the serine proteases and the thiol proteases.

The main metalloproteases involved in cartilage matrix degradation are collagenase, stromelysin and gelatinase.[22–24] Collagenase appears to be responsible for the breakdown of the collagen network in OA cartilage. An increased collagenase level has been identified *in situ* in human OA cartilage[25] as well as in the experimental dog OA model.[20] In addition, the collagenase level was found to correlate with the severity of OA cartilage lesions.[25] Stromelysin has been identified in human articular cartilage, and its level also correlates with the severity of OA lesions.[23,24] An increased level of collagenase and stromelysin mRNA has been found in OA chondrocytes,[26] and their levels enhanced in OA synovial fluid.[27] Furthermore, active stromelysin can mimic *ex vivo* and *in vitro* the breakdown of the proteoglycan monomer core protein, including HABR cleavage seen in OA.[17,18,28] Histochemical studies[24] have also revealed a correlation between the level of stromelysin and the degradation of pericellular proteoglycan. Degradation of types II and IX collagen was also reported to occur through stromelysin. Moreover, stromelysin may play a dual role in OA pathophysiology; on the one hand, by degrading matrix macromolecules itself and, on the other, by activating procollagenase.[29] The 92 kDa gelatinase is selectively expressed in fibrillated cartilage and is also likely involved in OA cartilage degradation.[22] The biological activity of metalloproteases is controlled by both physiological inhibitors and activators. At least two tissue inhibitors of metalloproteases (TIMP-1 and -2) are known to exist in humans. In OA cartilage, there is an imbalance between the synthesis and activity of TIMP and metalloproteases corresponding to a relative deficit in the amount of the inhibitor,[30,31] favouring an increased level of active metalloproteases and secondarily matrix degradation.

Serine and thiol dependent proteases, including the plasminogen activator (PA)/plasmin system and cathepsin B respectively, have both been suggested as possible activators of metalloproteases.[32,33] The plasmin system was shown to be involved in the activation of latent metalloproteases during *in vitro* studies, demonstrating that plasmin activated RA synovial collagenase.[33] However, a later study showed that complete collagenase activation requires, in conjunction with plasmin, the presence of active stromelysin.[29] To date, very few studies have addressed the involvement of PA/plasmin in OA pathophysiology. A recent report indicates that OA cartilage contained an increased level of plasmin associated with an increased synthesis in PA (urokinase type).[34] This study also revealed that a correlation exists between the level of plasmin and active collagenase in OA cartilage showing severe lesions.[34] Moreover, one of the major physiological inhibitors of plasminogen activators, PAI-1, was found to be markedly decreased in OA cartilage.[34] These findings, together with the increased level of PA, may partly explain the increased level of biologically active metalloproteases in OA tissue. Degradation of the extracellular matrix macromolecules often occurs in the pericellular area around the chondrocytes, where the matrix pH is in the acid range. At first, cathepsin D was thought to be the prime candidate for causing matrix degradation. Although cathepsin D is elevated in OA cartilage, it does not seem to be involved in cartilage resorption.[35] However, cathepsin B, another lysosomal enzyme, is likely to play an important role in cartilage degradation through its direct degradative effect on both collagen and proteoglycans, and also by activating metalloproteases.[36] Although cathepsin B is maximally active at pH 6.0, this enzyme can also exert proteolytic activity for a limited time at neutral pH.[36] As in several other human enzyme systems, the proteolytic effect of cathepsin B is regulated by specific protease inhibitors. Two such inhibitors, with molecular weights of 67 kDa and 13–16 kDa, have been found in articular cartilage.[37,38] It appears that the small inhibitors are forms of cystatin and the large inhibitor is a kininogen.[39,40] In OA cartilage, the cathepsin B level is increased, showing higher activity in cartilage lesions with a concomitant decrease in cysteine protease inhibitory activity.[37] This imbalance between cathepsin B and cysteine protease inhibitor levels may be an important contributing factor in OA cartilage degradation.

Rheumatoid arthritis (RA) is an autoimmune disorder characterized by a chronic, erosive synovitis of joints.[41] The cause of RA is unknown, and it is in fact possible that there are several causes for the disease. Infectious agents remain a focus of suspicion; however, there is no direct evidence for their involvement. An interrelationship between infectious agents, genetics and autoimmunity has also been suggested.[42]

In RA, the disability is due to damage to joint structures such as the capsule, ligaments and erosion of cartilage and bone. The initial pathologic changes in early RA happen at the synovial microvascular level with an activation and swelling of endothelial cells.[43] Plasma exudation which follows is reflected by the development of oedema in the subsynovial lining tissue. The cells in the synovial lining cell layer become activated, and their numbers are greatly increased. This lining consists of three cell populations: phagocytic cells of the monocyte–macrophage lineage, dendritic cell and fibroblast-like cells.[44] A small number of polymorphonuclear leukocytes are also observed at the superficial layer of the synovium. A large accumulation of mononuclear cells is seen around blood vessels and in the sublining synovial tissues. The sublining tissue is infiltrated with nodular collections of mononuclear cells, particularly around blood vessels. Activated T lymphocytes are predominant in focal aggregates and plasma cells at the periphery of the nodule. More diffuse collections of mononuclear cells consist of macrophages, T cells, B cells and plasma cells.[45] At chronic stages of the disease, the synovium becomes hypertrophic and villous projections of synovial tissue invade the joint cavity. The mass of inflammatory cells may invade over the surface of the articular cartilage (pannus) or may burrow into the subchondral bone. Joint destruction occurs predominantly in areas adjacent to the margin of the invading pannus.[46]

The destruction of the joint probably results from the production of an excessive amount of degradative enzymes. The major producer of enzymes is likely to be the synovial fibroblast at the synovial lining level. Polymorphonuclear leukocytes can also release several proteinases, and probably contribute to the proteolytic activity found in inflammatory synovial fluids.[47] Activation of neutrophils also results in the release of reactive oxidants which contribute to inflammation and injury of joints. The neutrophil proteases include elastase (serine protease), gelatinase and a collagenase

(metalloprotease); the latter is distinct from the collagenase synthesized by fibroblasts.[48,49] In the synovial lining cells, both the macrophages and fibroblast-like cells express collagenase and stromelysin.[50–52]

Interactions of cells with the cartilage matrix molecules are also important in the regulation of cellular activity in inflammation. The production of cytokines such as IL-1 is increased when mononuclear cells are exposed to several types of collagens such as types II, III and IX.[53,54] Fragments of type I collagen as well as other constituents of the bone matrix can stimulate production and release of IL-1 by monocytes.

Autoimmunity to cartilage matrix molecules may also play a role in cartilage destruction. Antibodies to cartilage collagens (types II, IX and XI) are commonly present in patients with rheumatic diseases.[55,56] The collagen fragments released from cartilage can possibly be recognized by the immune system as foreign proteins. Immune complexes containing antibodies to type II collagen have been observed in the superficial zone of articular cartilage in patients with RA.[56] The appearance of these antibodies occurs after articular cartilage damage has begun. The loss of cartilage may be accelerated by the deposition of immune complexes in the superficial layers of the cartilage, which favours invasion of the pannus.[57] Neuropeptides may play a role in the modulation of the inflammatory response.[58] Substance P, a peptide located in peripheral nerves, has been shown to induce the production of cytokines (IL-1, TNFα and IL-6) by mononuclear cells[59] and metalloproteases and prostaglandins by synovial fibroblasts.[60] This finding emphasizes the complexity of factors involved in the pathogenesis of RA.

It is likely that important interactions occur between monocyte/macrophages and synoviocytes resulting in the regulation of enzyme release, cytokine formation and cell proliferation. It is suggested that while immune mechanisms may initiate early stages of the disease, synoviocytes and macrophages are independently capable of maintaining a destructive phenotype and this may contribute to the chronic nature of RA.

### Role of cytokines in the destruction of joints

The spectrum of factors responsible for the altered function of synovium and cartilage in conditions such as OA and RA has not been fully defined. Prevailing theory dictates that the perpetuation of the above arthritic diseases is likely to be controlled by a complex cytokine network, in which three of them, IL-1, TNFα and IL-6 appear to be of major importance. These cytokines are soluble molecules that transmit information between cells. IL-1, TNFα and IL-6 have been detected in synovial fluid, synovium and cartilage from RA patients, and IL-1 and IL-6 in the latter tissues from OA patients. The major source of these cytokines in synovium is believed to be monocytes/macrophages; however, current evidence suggests that synovial lining cells (synoviocytes) also produce these cytokines. Findings suggest that IL-1 may be secreted by chondrocytes, whether this represents autocrine and/or paracrine stimulation is not yet known. It may be that this cytokine diffuses through the synovial fluid into the cartilage thus inducing chondrocytes to produce IL-1. Also, IL-6 is believed to be produced by chondrocytes.[61]

The three cytokines, IL-1, IL-6 and TNFα are synthesized and released as part of the response of their cells of origin to specific signals, and they influence the response and function of their target cells, largely by exerting a positive or negative influence on gene expression (Fig. 1). One important principle is reflected in their ability to cause multiple effects, overlapping and synergizing with other cytokines. The action of IL-1 and TNFα on joint tissues are multifaceted, with many different gene products being influenced either by stimulation or suppression. The association of these cytokines with tissue damage arises from their propensity to stimulate the proteolytic pathways of extracellular matrix degradation and, at the same time, subdue the synthetic pathways leading to new matrix formation.

It is likely that IL-1 is responsible for increasing the protease synthesis in diseased synovium, as metalloprotease production correlates with the severity of synovial inflammation[62] and the latter with the level of IL-1 in the synovial fluid[63]. The capacity of IL-1 produced by the inflamed synovium to stimulate the production of collagenase and PA by synovial fibroblasts has been well documented.[64–66] In synovium, protease inhibitors, such as TIMP or PAI-1, are either suppressed or unaffected by the cytokine IL-1. Mediators of the inflammatory process such as prostaglandin $E_2$ and IL-6 are both stimulated by IL-1. As synoviocytes secrete IL-1, it is tempting to speculate that autocrine stimulation may also play a role in the regulation of synoviocyte enzyme synthesis. IL-1 may also contribute to the fibrosis observed in arthritic synovium, as it increases the synthesis of types I and III collagens by synovial fibroblasts.[67,68]

Many of the effects of TNFα on synovium overlap with those of IL-1. In synovial tissue TNFα stimulates collagenase and prostaglandin $E_2$ production, displays synergism with other cytokines and, in certain circumstances, induces IL-1 production.

The exact role of IL-6 in arthritic synovium has not yet been clearly defined. The ability of IL-1 and TNFα to induce IL-6 protein and mRNA in synovial fibroblasts[69] suggests that this cytokine may be an important intermediate signal in the induction of other cellular responses to these cytokines. How-

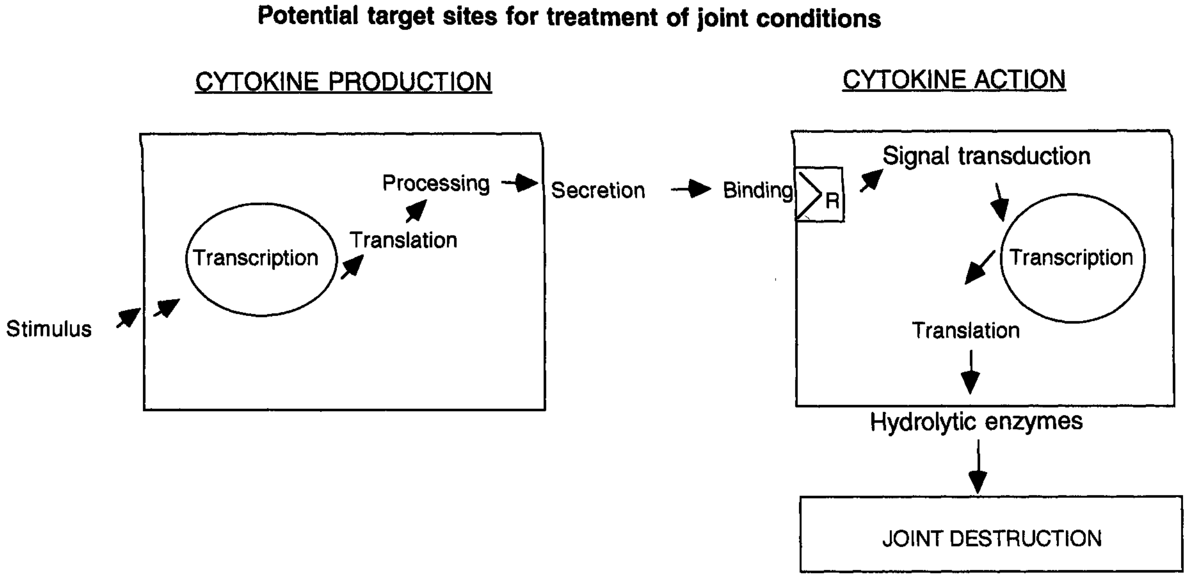

FIG. 1. Diagrammatic representation of potential sites at which the destructive activities of cytokines can be modulated. The biological effects of cytokines can be modulated at points associated with cytokine production, including transcription, translation, secretion and degradation; or at points associated with cytokine action, including inhibition of receptor binding, signal transduction or inhibitors of hydrolytic enzymes.

ever, IL-6 has no direct effects on the synthesis of proteases, prostaglandins or matrix proteins, but stimulates synthesis of TIMP.[70] It is suggested that IL-6, by its *in vivo* activation of B cells, may contribute to the immunologic phenomenon; its role in inflammation may be via regulation of changes in the concentrations of acute phase proteins.

Another important effect of cytokines on the development of synovitis is their contribution to the expression of adhesion molecules, which in turn control the accumulation of leukocytes. Evidence exists that IL-1 and TNFα are involved in the early events affecting the joints by their contribution to the migration of cells to the site of inflammation and their stimulation of a variety of responses in endothelial cells. At present, four families of adhesive molecules have been described:[71] the immunoglobulin supergenes, the integrins, the cadherins and the syndecans. The cytokines IL-1 and TNFα were recently shown to upregulate the expression of some of the members of the first family, the ICAM, present on fibroblast and endothelial cells.[72] It is likely that increased ICAM expression plays an important initial step in the binding of leukocytes to synovial endothelial cells in RA joints[73] and facilitates the subsequent entry of these cells into the inflammatory synovial fluid. Yet no effect by these cytokines has been demonstrated on members of the other adhesion molecule families. In synovial fluid, as well as in the synovial tissues, polymorphonuclear leukocytes are attracted and are believed to play a role in tissue destruction by their release of cytokines and degrading proteases. Evidence is mounting that a new cytokine superfamily, known as the intercrine (or chemokine), the members of which have the ability to mediate the recruitment of leukocytes, is likely to play an important role in inflammation.[74,75] As these intercrines exhibit differing patterns of specificity for various leukocyte populations, this makes them attractive candidates as important components of the inflammatory process. Members of this family are classified as two groups according to the position of the first two cysteines in the conserved motifs. Among the first group are IL-8 and MCP-1; RANTES belongs to the second group. The members of the first group appear to be chemotactic for neutrophils, but not for mononuclear leukocytes, whereas the second group members attract mostly mononuclear cells and granulocytes. Recently, it was shown the MCP-1 and IL-8 are expressed in monocyte/macrophages, fibroblasts and endothelial cells in response to the cytokines TNFα and IL-1.[76–81] Similarly, RANTES expression in fibroblasts was also found to be enhanced by these two latter cytokines.[82] Although they are present in the synovial environment of RA and OA,[81,83–85] their diversity of function and their mutual interaction have led to considerable confusion. However, it is important to recognize that they are generated only after specific cell–cell interaction and their activity is limited to the microenvironment immediately surrounding the cell that produced it.

The action of cytokines is not unique to synovial membranes, they also compromise articular cartilage functions. In articular cartilage, IL-1, TNFα and IL-6, appear among the known cytokines to play a major role in the pathological process. In addition to the above-mentioned effects on the synovium, IL-1 suppressed the synthesis of collagen types characteristic of hyaline cartilage (type II and type IX), while promoting the synthesis of these collagen types characteristic of fibroblasts[86] (type I and type III), thereby causing the decreased repair of cartilage matrix. The synthesis of the aggregating proteoglycan (aggrecan) characteristic of hyaline cartilage is also suppressed, although those proteoglycan molecules that are synthesized appear to undergo normal post-translational processing.[87] It has been suggested recently that the effect of IL-1 on the inhibition of proteoglycan synthesis may be mediated by IL-6 in human cartilage, and that IL-1 induces the synthesis and secretion of IL-6 by human chondrocytes.[88,89] Interestingly, the action of IL-1 on different connective tissue cells does not always produce the same response, i.e. stimulation or suppression. For example, in contrast to the effect of IL-1 on chondrocytes, IL-1 stimulates glycosaminoglycan synthesis by synovial fibroblasts.[90] This is probably due to the differential expression of aggrecan in synovial fibroblasts and chondrocytes. In cartilage, by far the greatest proportion of glycosaminglycan is synthesized as aggrecan. However, in synovium, aggrecan is not significantly expressed, but decorin is abundant, and the latter gene is upregulated by IL-1.[91] The effects of IL-1 on cartilage proteins are not limited to inhibition of synthesis, as prostaglandin $E_2$ production is stimulated by this cytokine.[92] This stimulation of prostaglandin production is of particular interest because of the role it may play in exacerbating joint inflammation, stimulating bone resorption and modulating the immune response. Finally, IL-1 may be involved in osteophyte formation, by stimulating the proliferation of human osteoblast-like cells causing increased bone formation.

In cartilage, TNFα appears to induce many effects analogous to those generated by IL-1, although the former is generally less potent in its effect than either form of IL-1.[93] Although the effect of TNFα on chondrocytes has been less well studied than that of IL-1, it is clear that TNFα, like IL-1, can stimulate the production of proteolytic enzymes such as collagenase, stromelysin, elastase and PA, as well as prostaglandins $E_2$ and IL-6. TNFα also has, however, no effect on TIMP production by articular chondrocytes. TNFα also modulates the synthesis of cartilage matrix and, like IL-1, suppresses aggregating proteoglycan synthesis[94] and selectively decreases the production of cartilage type I and type II collagens. It suppresses the expression of type II collagen, but increases type I and III collagen gene expression.[95]

In contrast to its detrimental role of inhibiting proteoglycan synthesis,[88] IL-6 does not appear to influence the production of the metalloproteases, either alone or in combination with IL-1.[96,97] Indeed, it appears to stimulate the production of TIMP.[70,97] In this manner, IL-6 production would counteract the degradative potential of IL-1. In cartilage, the involvement of IL-6 in the proliferation of chondrocytes as well as clones is plausible, as this cytokine has been shown to enhance human OA chondrocyte proliferation.[98]

Finally, the process involved in the inhibition/activation process of metalloproteases in arthritic joint tissues could very well be modulated by IL-1. For instance, the imbalance in the TIMP-1 and metalloproteases levels[31] in articular cartilage may be mediated by IL-1, as *in vitro* experiments showed that increasing concentrations of IL-1 produced decreased TIMP-1 synthesis in parallel with increased metalloprotease synthesis in articular cartilage and chondrocytes.[30,34] PA synthesis is also modulated by IL-1. *In vitro* stimulation of cartilage chondrocytes with IL-1 showed a dose dependent increase of the PA, concomitant with a sharp decrease in PAI-1 synthesis.[99–101] The potent inhibitory effect of IL-1 on PAI-1 synthesis, in combination with a stimulatory effect on PA synthesis, is a powerful mechanism for regulation of the generation of plasmin and metalloprotease activation. In addition to its role as an enzyme activator, plasmin may also be involved in cartilage matrix degradation by direct proteolysis of the proteoglycan monomer.[102]

### Potential stages for cytokine reduction

Cytokine production is associated with disturbances of homeostasis ranging from acute conditions such as sepsis to chronic connective tissue disorders such as RA and OA. Distinctly different strategies of cytokine modification are required for blocking the effects of cytokines produced locally and chronically in joint conditions compared with those employed for short periods when relatively high systemic levels are produced, as in sepsis.[103] Local elevation of cytokines in joints during RA and OA may result in minimal increases in systemic blood levels. Thus TNα, IL-1 and IL-6, if detectable, are much lower than in sepsis, making direct measurement of cytokines difficult.[104]

Cytokine production can be assessed indirectly by their biological effects. One of the best measured sequelae of cytokine production and action is a marked change in the pattern of hepatic synthesis of plasma proteins. Plasma proteins whose synthesis is responsive to cytokines (or secondarily to inflammation, trauma or sepsis) are known as acute phase proteins or acute phase reactants. Two inducible acute phase proteins, in particular, serum amyloid A (SAA) and C-reactive protein (CRP), whose synthesis is regulated by synergism of IL-1 and IL-6,[105–107] have prognostic value in the clinical management of arthritis. The concentrations of IL-1 and IL-6 in plasma of RA patients are considerably lower than that predicted by *in vitro* studies to be required for stimulation of SAA and CRP production. Thus the nature of the signals to liver for acute phase protein synthesis are not completely understood and are the subject of active investigation in several laboratories. One possibility is that the signals from inflamed joints may not be IL-1 and/or IL-6, but rather TNFα or another signal that stimulates IL-1 and/or IL-6 production by Kupffer cells. Hepatocytes adjacent to the cytokine-producing Kupffer cells are then able to respond. A second possibility is that inflammatory cells may travel from inflamed joints to the liver where they come into contact with hepatocytes and then locally produce the cytokines by which hepatic cells are stimulated. A third possibility is that mediators that are currently unknown may travel to the liver to stimulate SAA and CRP synthesis. Although the exact pathway by which inflamed joints signal changes in hepatic protein synthesis is unclear, it has been recognized for more than 20 years that elevated concentrations of acute phase proteins are associated with a poor clinical prognosis and more recently, that reduction of disease activity by anti-rheumatic therapy is accompanied by reduced plasma concentrations of acute phase proteins such as SAA and CRP.[108–113] Measurement of acute phase proteins thus provides a strategy by which therapies which inhibit cytokine synthesis or action in the treatment of joint conditions can be monitored.

Several cytokines have been detected in high concentrations in RA synovial fluid and synovial membrane including IL-1, IL-6, TNFα,[114–123] GM–CSF,[124] IL-8[81] and TGFβ.[118,125] IL-1, TNFα and IL-6 are not only found in RA synovium, but have been detected in serum and synovial fluid of arthritis patients.[126,127] Both IL-1 and TNFα are thought to play a significant role in the pathogenesis of arthritis, but cannot totally account for the pathology of RA and OA.[128] The leukocytic infiltration into the synovial lining and the synovial fluid is thought to be due to production of other mediators such as GM–CSF and IL-8, secondary to IL-1 or TNFα. Cytokine production and action may play a role in arthritic joint conditions in a number of ways, including disease induction, chronicity and exacerbation. Thus cytokine blocking agents will be of broad value in treating joint conditions. There are several stages at which cytokine production can be blocked, including transcription, translation, secretion and action. Agents that reduce the synthesis and action of cytokines known to be involved in joint disease will be reviewed briefly in the following sections.

*Tumour necrosis factor-α*: RA is characterized by cellular activation and TNFα production.[118] TNFα, originally identified for its anti-tumour activity and subsequently for its cachectic activity, is known to influence inflammation and cellular immune responses.[129–133] Transgenic mice that produce large amounts of human TNFα develop arthritis,[134] and TNFα administered into knee joints of animals causes an acute arthritis.[135,136] TNFα, mainly derived from monocyte/macrophage derived cells, signals its responses through two distinct cell surface receptors, TNF-R55 and TNF-R75.[137–139] While TNF-R55 and TNF-R75 differ in the primary structures of their cytoplasmic domains, both are thought to bring about cellular alterations by altering gene expression through activation of various nuclear transcription factors, which act as a link between cell membrane and nucleus. TNF-R are localized in synovial tissue and the cartilage–pannus junction in RA patients to a much greater extent than in OA patients.[140]

The single 3 000 bp TNFα gene consisting of four exons is on chromosome 6. Induction of TNFα synthesis is under both transcriptional and post transcriptional regulation.[141] Mature TNFα, 17 kDa, 157 amino acids in length, is derived from a 26 kDa transmembrane precursor protein[142] by cleavage of a 76 amino acid region from the amino terminus; the amino terminal region is thought to anchor the TNFα precursor in the plasma membrane. Native biologically active TNFα is a trimer with a three-dimensional structure similar to viral capsids.

Because TNFα is produced locally at sites of inflammation and plays a pivotal role in the cytokine network, it is desirable to prevent TNFα production at the earliest stages. One promising technique is the application of ribozymes, which have been designed to cleave target viral RNAs including HIV-1, and are considered to show promise as *in vivo* therapeutic agents if obstacles to co-localization with their substrates can be overcome.[143] In particular, it has been thought that the compartmentalization of RNAs in cells would limit the efficacy of ribozymes due to reduced diffusion.[144] Local administration of ribozymes specific for TNFα to the joint has been proposed,[145] and preformed ribozymes have been shown in a model system to reduce TNFα mRNA and production by 90% and 85%, respectively.[145]

Several approaches have been used to inhibit the interaction of TNFα with its receptors. Suramin, which has been used experimentally to block ligand–receptor interactions, has been found to inhibit the biological activity of human TNFα through direct action on the ligand, i.e. by dissociation of the quaternary structure of TNFα.[146,147] Recently, a chimeric monoclonal antibody to TNFα was used to treat patients with RA[148] with significant clinical improvements and changes in laboratory parameters including decreased CRP, SAA and IL-6 concentrations. Since CRP and SAA both appear to be stimulated by synergism of IL-1 and IL-6 and TNFα alone is less potent, this finding is consistent with the concept that TNFα is an early mediator that acts by way of IL-1 and IL-6.[3]

TNFα inhibitory proteins identified in serum and urine, were found to be fragments of the extracellular portions of TNF receptors.[149–153] These TNFα binding proteins can reach concentrations of greater than 2 ng/ml and are thought to neutralize TNF bioactivity or to act as reservoirs from which TNF can slowly be released. Recombinant soluble TNF receptors prevent development of experimental collagen arthritis[154] as do anti-murine TNFα antibodies.[154,155]

*Interleukin-1:* As discussed above, IL-1 is a mediator of arthritis with activity at many points in the pathogenesis of the disease, e.g. capable of inducing synthesis of metalloproteinases implicated in cartilage loss, adhesion molecules necessary for the migration of inflammatory cells into inflamed tissue, and synthesis of cytokines such as IL-6, IL-8 and GM–CSF. Animal studies have shown that IL-1 directly injected into knee joints will cause arthritis, will induce a flare of quiescent, pre-existing arthritis and will increase incidence and severity of arthritis in prearthritic animals.[156–158]

Two distinct forms of human IL-1 have been characterized, IL-1α and IL-1β (reviewed in References 159 and 160). The two forms have ~27% amino acid identity and are the products of separate genes. The primary IL-1 gene products are 31 kDa proteins of about 270 amino acids and are precursors of the active species designated IL-1α (159 amino acid residues) and IL-1β (153 amino acid residues). The IL-1 precursors lack classical hydrophobic leader sequences and the 17 kDa mature IL-1 carboxyl peptides are formed by action of specific proteases upon secretion.

The human IL-1α gene is 10 kb in length and the IL-1β gene is ~7 kb long. Both are on chromosome 2 and are comprised of seven exons and six introns. Pro-IL-1 gene expression is regulated both transcriptionally and post-transcriptionally.[161,162] The IL-1 genes are under specific transcriptional control which is influenced by cell type and the inducing agent, and modulated by other cytokines. The differential expression of IL-1α and IL-1β may be explained in part by the marked structural differences between the two promoters.[163]

The biological properties of IL-1 are similar to and overlap those of TNF. In some situations, potentiation or synergism between IL-1 and TNF occurs, an effect that seems to derive from signal transducing molecules rather than upregulation of receptors, since IL-1 down-regulates TNF receptors.[164,165]

As reviewed by Fenton,[162] transcription, translation and release of IL-1 are distinct, dissociable processes. Secretion and processing of pro-IL-1β appear to be linked. There are redundant mechanisms for controlling IL-1 production, and, once produced, there are multiple mechanisms for regulating the effects of IL-1 (reviewed in Reference 160). These include the number of and the presence of receptors on target cells, potential production of soluble receptors and synthesis of inhibitors.

There are two Rs for IL-1, the 80 kDa type I and the 60 kDa type II. In type II IL-1R, the cytoplasmic domain portion is shorter than for type I IL-1R. Antibodies to the type I IL-1R block the biological effects of IL-1 whereas type II IL-R appears to be a decoy receptor.[166] In contrast to antibodies to TNF receptors, antibodies to IL-1Rs have not yet been associated with agonist activities.[103,150]

Soluble shed type I IL-1R has not been described in nature; type II IL-1R is shed.[166,167] The extracellular domain of type II IL-1R has been cloned and expressed and shown to bind both forms of IL-1. In terms of cytokine reduction, an advantage that sIL-1R (or antibodies to IL-1) would seem to have over IL-1RA is that quantities that are only stoichiometic to IL-1 would be required to reduce IL-1 actions, rather than the 1 000-fold excess that would seem to be required of a competitive inhibitor, as studies in animal models have indicated.[168] The naturally occurring IL-1RA is induced at high levels and competes with IL-1 for binding to its receptor,[126,169] thereby serving to buffer the intensity of an inflammatory response. Cloned IL-1RA inhibits the activities of IL-1α and IL-1β.[126,141] Production of IL-1 and IL-1RA are regulated differently (reviewed in Reference 103).

IL-1 is a mediator of rheumatoid synovitis and there are several stages at which its action can be blocked. Transforming growth factor-β (TGFβ) is known to counteract the effects of IL-1, perhaps by reducing the number of IL-1 receptors or by stimulation of IL-1RA release.[170] In animal models of arthritis, IL-1RA has been shown to inhibit the flare of streptococcal arthritis induced by streptococcal cell walls, and anti-murine IL-1 antibody effectively reversed the inhibition of proteoglycan synthesis and loss of cartilage that accompany monoarticular antigen arthritis.[171] An initial study of subcutaneously administered IL-1RA in RA has been reported[172] with greater than 50% reduction in swollen joints and CRP concentration.

Antisense oligonucleotides form duplexes with their corresponding sense mRNAs and prevent transcription.[173] Antisense IL-1α inhibits the programmed cell apoptosis of cultured endothelial cells.[174] Antisense inhibition of IL-1R expression is actively being pursued. Because of difficulties in transport, uptake and targeting of antisense oligonucleotides,[175] topical or local administration such as in the cases of psoriatic arthritis would seem the most promising for initial investigation.

Synovial fibroblasts have been transfected with IL-1RA mRNA. Cells constitutively producing IL-1RA have been injected into rabbit knee joints and shown to block an IL-1 induced synovitis.[176] IL-1β production uniquely requires an enzyme termed pro-IL-1β

converting enzyme (ICE). Specific inhibition of ICE appears to be a promising approach to arthritis treatment in situations in which only IL-1β is involved. However, it remains to be determined if there is redundancy of IL-1α and IL-1β in the pathophysiology of arthritis.

*Interleukin-6:* The IL-6 gene is located on chromosome 7 and consists of five exons; expression is induced by agents such as IL-1 and TNF to yield a multifunctional cytokine that acts on many different types of cells.[103,177,178] IL-6 signals target cells through 80 kDa cell surface receptors[179] which as a complex, IL-6/R, interacts with a 130 kDa protein to initiate signal transduction.[180] Both the 80 kDa R and the 130 kDa signal transducing protein are subject to regulation by inflammatory mediators. For example dexamethasone (Dex) stimulates, while high IL-6 levels down-regulate the 80 kDa IL-6R. In contrast, IL-6, Dex and the combination stimulate the 130 kDa signal transducing protein. A soluble derivative of the 80 kDa receptor found in urine,[181] is thought to be generated by limited proteolysis (shedding) of the 80 kDa plasma membrane IL-6R.[179] The soluble IL-6 receptor consists of the extracellular region only and lacks the transmembrane and cytoplasmic region; soluble IL-6R/IL-6 complex acts as an agonist for *in vitro* acute phase protein synthesis. Thus in situations of sustained high IL-6 and down-regulated IL-6R, the soluble IL-6R may have an important physiologic role in modulating the activity of IL-6. The IL-6 receptor subunits belong to a superfamily which includes LIF, oncostatin M, CNTF and GM–CSF.[179] One IL-6 action, fever, is blocked by cyclooxygenase inhibitors indirectly through increased release of arachidonic acid.[103] Soluble p80R for IL-6 enhances activity.[181,182]

### Cytokine reducing effects of anti-arthritic drugs

The scope, target mechanisms and effectiveness of anti-rheumatic drugs in the pre-cytokine era were thoroughly reviewed by Bonta and colleagues.[183] The use of cyclooxygenase inhibitors to inhibit the production of prostaglandins has been a major element in the clinical management of arthritis for about 100 years, while our understanding of the role of prostaglandins in inflammation has been developed over the past 20 years.

*Non-steroidal anti-inflammatory drugs:* The pro- and anti-inflammatory action of PGE has been recognized for a number of years.[183] Prostaglandins affect inflammation by several mechanisms. They alter blood flow through inflamed areas, potentiate capillary leakage, potentiate sensory fibre pain transmission and, by inducing intracellular cAMP, regulate synthesis of proteins with cAMP regulatory elements, such as TNFα and IL-2. NSAIDs (aspirin, indomethacin, fentiazac, naproxen, piroxicam) increase TNF and IL-2, cytokines whose synthesis is under regulation by $PGE_2$;[184–187] however, acute phase protein concentration is usually unaffected by NSAIDs. $PGE_2$ and other prostanoids inhibit interferon-γ production whereas they are required for PA synthesis.[186,188–189] NSAIDs appear to act by inhibition of the synthesis of prostaglandins. Recently, two cyclooxygenase enzymes have been identified. The constitutive enzyme is called Cox-I and the second enzyme, termed Cox-II, is induced by cytokines produced at the sites of inflammation. Selective Cox II inhibitors are being designed in the hope of greater selectivity in arthritic disease and decreased gastrointestinal side effects.[191]

*Glucocorticoids:* Glucocorticoids, like insulin, exert, at concentrations usually found in plasma, direct regulatory effects on inflamed areas such as joints.[192] Steroid analogues of cortisol, the major active glucocorticoid in humans, such as hydrocortisone and dexamethasone, inhibit release of arachidonic acid from phospholipids of cell membranes by inhibiting the activation of phospholipase $A_2$. Glucocorticoids inhibit collagen synthesis, and are well known inhibitors of IL-1 production acting at both transcriptional[193] and post-transcriptional levels.[194–196] Glucocorticoids have also been reported to have a general regulatory effect, inhibiting production and activity of cytokines such as IL-2, -4, -6, -8, GM–CSF and TNFα.[195–201] IL-10 and dexamethasone inhibit IL-1 and TNF production from LPS-stimulated monocytes.[194,195,202–206]

*Immunomodulators and immunosuppressive agents:* RA has been classified as an autoimmune disease since the pathologic manifestations of RA resemble a misdirected immune response. T cells have been implicated in establishment of chronic arthritic disease. Since antigen or mitogen stimulation of T cells leads to increased expression of IL-2R, it has been suggested that removal of activated IL-2R expressing T cells during active disease could result in selective depletion of those T cells involved in the disease process. Furthermore, since RA probably results from specific sets of antigen-responsive cells, selective inhibition of these sets of T cells could provide therapy that does not suppress general immune function. A trial of anti-IL-2R has been reported.[207] Chimeric toxin-IL-2 fusion proteins may be useful in RA treatment. A recent study has used oral chicken collagen as treatment in an animal model of arthritis, a treatment based on our knowledge that oral exposure to antigen can result in long-lasting tolerance to that antigen.[208] Results to date are limited to animal models for which the inciting antigen is defined.

Immunomodulatory and/or cytotoxic plant alkaloids, are under investigation for their capacity to control the production and action of pro-inflammatory cytokines such as IL-1 and TNF. Compounds derived from extract of plant roots have been tested including tetrandine[209] and extracts of *Trypterygium wilfordii* Hook F[210] including triptolide and tripdiolide.[211] These compounds are frequently potent in their activity, but remain to be extensively characterized.

*Others:* A synthetic analogue of fumagillin, AGM 1470 has been shown to inhibit experimental collagen arthritis, presumably by inhibiting endothelial cell growth and thus neovascularization.[212,213] In animals models of OA, tetracycline shows promise for treatment.[214,215]

Tenidap and IX-207-887, which have advanced into clinical trials, have been shown to inhibit IL-1 synthesis and/or action.[216–220] IL-1R in OA are upregulated, whereas TNF-R occur at only low levels[221] and tenidap has been shown to down-regulate IL-1R.[222] In *in vitro* studies, tenidap has been shown to inhibit IL-6 production by PBMCs to an even greater extent than IL-1 is inhibited; furthermore tenidap treatment has been shown to lower CRP and SAA concentrations.[217,223] Tenidap treatment has recently been shown to lower CRP concentrations in OA patients.[113] SKF-86,002 and chloroquine inhibit IL-1 synthesis and/or action.[196,224] L-709,049 and SK&F 86002 interfere with IL-1β secretion.[225] Although there are no reports of biological or synthetic IL-8 antagonists, quinolylmethoxyphenylamine (ETH 615) inhibition of IL-8 biosynthesis has been reported.[226]

### Summary and future perspectives

Based on animal studies, T cell involvement in RA may play a critical role in initiation and perpetuation of disease. As studies with cyclosporin A suggest, T cell selective agents provided one promising avenue of therapy. There is also little question that proteases act directly to cause cartilage destruction in both RA and OA. Selective inhibition of one or more of these enzymes may provide a second avenue for treatment of RA and OA. The involvement of cytokines, particularly IL-1 and TNF, has been strongly supported in animal models of arthritis by therapy using selective anti-cytokine antibodies, anti-cytokine receptor antibodies, cytokine receptor antagonist proteins, and soluble and chimeric cytokine receptor molecules. These studies suggest that IL-1 and TNF may play additive, if not synergistic, roles, since inhibition of either IL-1 or TNFα provides therapeutic benefit. Because of the plethora of potential pathological consequences of cytokine elevation, pharmacologic regulation of IL-1 and TNFα remain primary targets for treatment of arthritis. Early clinical trials of very selective biological compounds support the therapeutic value of cytokine inhibitors. However, the results of long-term clinical trials of selective pharmacological cytokine inhibitors will be required to define precisely the pathological roles of cytokines in arthritis and the value of anti-cytokine therapy.

### References

1. Harris ED. Rheumatoid arthritis: pathophysiology and implications for therapy. *N Engl J Med* 1990; **322**: 1277–1289.
2. Ziff M. Rheumatoid arthritis: its present and future. *J Rheumatol* 1990; **17**: 127–133.
3. Brouckaert P, Libert C, Everaerdt B, Takahashi N, Cauwels A, Fiers W. Tumor necrosis factor, its receptors and the connection with interleukin 1 and interleukin 6. *Immunobiol* 1993; **187**: 317–329.
4. Akira S, Hirano T, Tagia T, Kishimoto T. Biology of multifunctional cytokines: IL-6 and related molecules (IL-1 and TNF). *FASEB J* 1990; **4**: 2860-2867.
5. Herman JH, Appel AM, Khosla RC, Hess EV. *In vitro* effect of select nonsteroidal antiinflammatory drugs on the synthesis and activity of anabolic regulatory factors produced by osteoarthritic and rheumatoid synovial tissue. *J Rheumatol* 1989; **16**: 75–81.
6. Byer PD, Bayliss MT, Maroudas A, et al. Hypothesizing about joints. In: Maroudas A, Holborow EJ, eds. *Studies in Joint Disease 2.* London: Pitman Co, 1983; 241–276.
7. Williams CJ, Jimenez SA. Heredity, genes and osteoarthritis. In: Moskowitz RW, ed. *Rheumatic Disease Clinics of North America: Osteoarthritis.* Philadelphia: W.B. Saunders, 1993; 523–544.
8. Dieppe P. Osteoarthritis and related disorders. In: Weatherall L, JGG, eds. *Oxford Textbook of Medicine.* New York: Oxford University Press, 1987; 1676–1684.
9. Mankin HJ, Brandt KD, Shulman LE. Workshop on etiopathogenesis of osteoarthritis: proceedings and recommendations. *J Rheumatol* 1986; **13**: 1130–1160.
10. Hamerman D. The biology of osteoarthritis. *N Engl J Med* 1989; **320**: 1322–1330.
11. Pelletier JP, Howell DS. Etiopathogenesis of osteoarthritis. In: McCarty DJ, Koopman WJ, eds. *Arthritis and Allied Conditions.* Philadelphia: Lea & Febiger, 1993; 1723–1734.
12. Howell DS. Pathogenesis of osteoarthritis. *Am J Med* 1986; **80**: 24–28.
13. Mankin HJ, Dorfman H, Lippiello L, Zarins A. Biochemical and metabolic abnormalities in articular cartilage from osteo-arthritic human hips. II. Correlation of morphology with biochemical and metabolic data. *J Bone Joint Surg* 1971; **53A**: 523–537.
14. Sokoloff L. Osteoarthritis as a remodeling process. *J Rheumatol* 1987; **14**: 7–10.
15. Inerot S, Heinegard D, Audell L, Olsson SE. Articular cartilage proteoglycans in aging and osteoarthritis. *Biochem J* 1978; **169**: 143–156.
16. Mankin HJ. The reaction of articular cartilage to injury and osteoarthritis. *N Engl J Med* 1974; **291**: 1285–1292.
17. Martel-Pelletier J, Pelletier JP, Malemud CJ. Activation of neutral metalloprotease in human osteoarthritic knee cartilage: evidence for degradation in the core protein of sulphated proteoglycan. *Ann Rheum Dis* 1988; **47**: 801–808.
18. Tyler JA. Chondrocyte-mediated depletion of articluar cartilage proteoglycans *in vitro. Biochem J* 1985 **255**: 493–507.
19. Sandy JD, Neame PJ, Boynton RE, Flannery CR. Catabolism of aggrecan in cartilage explants. Identification of a major cleavage site within the interglobular domain. *J Biol Chem* 1991; **266**: 8683–8685.
20. Pelletier JP, Martel-Pelletier J, Altman RD, *et al.* Collagenolytic activity and collagen matrix breakdown of the articular cartilage in the Pond–Nuki dog model of osteoarthritis. *Arthritis Rheum* 1983; **26**: 866–874.
21. Livne E, von der Mark K, Silbermann M. Morphologic and cytochemical changes in maturing and osteoarthritic articular cartilage in the temporomandibular joint of mice. *Arthritis Rheum* 1985; **28**: 1027–1038.
22. Mohtai M, Smith RI, Schurman DJ, *et al.* Expression of 92kD type IV collagenase/gelatinase (gelatinase B) in osteoarthritis cartilage and its induction in normal human articular cartilage by interleukin-1. *J Clin Invest* 1993; **92**: 179–185
23. Dean D. Proteinase-mediated cartilage degradation in osteoarthritis. *Sem Arthritis Rheum* 1991; **20**: 2–11.
24. Pelletier JP, Martel-Pelletier J, Cloutier JM, Woessner J Jr. Proteoglycan-degrading acid metalloprotease activity in human osteoarthritic cartilage, and the effect of intraarticular steroid injections. *Arthritis Rheum* 1987; **30**: 541–548.
25. Pelletier JP, Martel-Pelletier J, Howell DS, Ghandur-Mnaymneh L, Enis JE, Woessner JF. Collagenase and collagenolytic activity in human osteoarthritic cartilage. *Arthritis Rheum* 1983; **26**: 63–68.
26. Zafarullah M, Martel-Pelletier J, Cloutier JM, Gedamu L, Pelletier JP. Expression of c-fos, c-jun, jun-b, metallothionein and metalloproteinase genes in human chondrocytes. *FEBS Lett* 1992; **306**: 169–172.
27. Lohmander LS, Hoermer LA, Lark MW. Metalloproteinase, tissue inhibitor, and proteoglycan fragments in knee synovial fluid in human osteoarthritis. *Arthritis Rheum* 1993; **36**: 181–189.
28. Campbell IK, Roughley PJ, Mort JS. The action of human articular cartilage metalloprotease on proteoglycan and link protein. Similarities between products of degradation *in situ* and *in vitro. Biochem* J 1986; **237**: 117–122.
29. Murphy G, Cockett MI, Stephens PE, Smith BJ, Docherty AJ. Stromelysin is an activator of procollagenase. A study with natural and recombinant enzymes. *Biochem J* 1987; **248**: 265–268.

30. Martel-Pelletier J, McCollum R, Fujimoto N, Obata K, Cloutier J-M, Pelletier JP. Excess of metalloprotease over TIMP may contribute to cartilage degradation in osteoarthritis and rheumatoid arthritis. *Lab Invest* 1994; (in press).
31. Dean DD, Martel-Pelletier J, Pelletier JP, Howell DS, Woessner J Jr. Evidence for metalloproteinase and metalloproteinase inhibitor (TIMP) imbalance in human osteoarthritic cartilage. *J Clin Invest* 1989; **84**: 678–685.
32. Eeckhout Y, Vaes G. Further studies on the activation of procollagenase, the latent precursor of bone collagenase. Effects of lysosomal cathepsin B, plasmin and kallikrein, and spontaneous activation. *Biochem J* 1977; **166**: 21–31.
33. Werb Z, Mainardi CL, Vater CA, Harris E Jr. Endogenous activation of latent collagenase by rheumatoid synovial cells. *N Engl J Med* 1977; **296**: 1017–1023.
34. Martel-Pelletier J, Faure MP, McCollum R, Mineau F, Cloutier JM, Pelletier JP. Plasmin, plasminogen activators and inhibitor in human osteoarthritic cartilage. *J Rheumatol* 1991; **18**: 1863–1871.
35. Hembry RM, Knight CG, Dingle JT, Barrett AJ. Evidence that extracellular cathepsin D is not responsible for the resorption of cartilage matrix in culture. *Biochim Biophys Acta* 1982; **714**: 307–312.
36. Mort JS, Recklies AD, Poole AR. Extracellular presence of the lysosomal proteinase cathepsin B in rheumatoid synovium and its activity at neutral pH. *Arthritis Rheum* 1984; **27**: 509–515.
37. Martel-Pelletier J, Cloutier JM, Pelletier JP. Cathepsin B and cysteine protease inhibitors in human osteoarthritis. *J Orthop Res* 1990; **8**: 336–344.
38. Killackey JJ, Roughley PJ, Mort JS. Proteinase inhibitors of human articular cartilage. *Collagen Rel Res* 1983; **3**: 419–430.
39. Barrett AJ, Rawlings ND, Davies ME, Machleidt W, Salvesen G, Turke J. Cysteine protease inhibitors of the cystatin super family. In: Barrett AJ, Salvesen G, eds, *Proteinase Inhibitors*. Amsterdam: Elsevier, 1986; 515–569.
40. Legris F, Martel-Pelletier J, Pelletier JP, Colman R, Adam A. An ultrasensitive chemiluminoenzymatic assay for the quantification of human tissue kininogen. *J Immunol Methods* 1994; (in press).
41. Harris E Jr. Rheumatoid arthritis: etiology and pathogenesis. In: Kelly WH, Harris ED Jr, Ruddy S, Sledge CB, eds, *Textbook of Rheumatology*. Philadelphia: W.B. Saunders, 1993; 833–873.
42. Albani S, Tuckwell JE, Esparza L, Carson DA, Roudier J. The susceptibility sequence to rheumatoid arthritis is a cross-reactive B cell epitope shared by the *Escherichia coli* heat shock protein dnaj and the histocompatibility leukocyte antigen DRB 10401 molecule. *J Clin Invest* 1992; **89**: 327–331.
43. Schumacher HR. Synovial membrane and fluid morphologic alterations in early rheumatoid arthritis: microvascular injury and virus-like particles. *Ann NY Acad Sci* 1975; **256**: 39–64.
44. Burmester GR, Jahn B, Rohwer P, Zacher J, Winchester RJ, Kalden JR. Differential expression of Ia antigens by rheumatoid synovial lining cells. *J Clin Invest* 1987; **80**: 595–604.
45. Haraoui B, Pelletier JP, Cloutier JM, Faure MP, Martel-Pelletier J. Synovial membrane histology and immunopathology in rheumatoid arthritis and osteoarthritis. *Arthritis Rheum* 1991; **34**: 153–163.
46. Krane SM. Mechanisms of tissue destruction in rheumatoid arthritis. In: McCarty DJ, Koopman WJ, eds. *Arthritis and Allied Conditions—Textbook of Rheumatology*. Philadelphia: Lea and Febiger, 1993; 1723–1734.
47. Mainardi CL, Hasty DL, Seyer JM, Kang AH. Specific cleavage of human type III collagen by human polymorphonuclear leukocyte elastase. *J Biol Chem* 1980; **255**: 12006–12010.
48. Hasty KA, Pourmotabbed TF, Goldberg GI, *et al.* Human neutrophil collagenase. A distinct gene product with homology to other matrix metalloproteinases. *J Biol Chem* 1990; **265**: 11421–11424.
49. Hibbs MS, Hasty KA, Seyer JM, Kang AH, Mainardi CL. Biochemical and immunological characterization of the secreted forms of human neutrophil gelatinase. *J Biol Chem* 1985; **260**: 2493–2500.
50. Firestein GS, Paine MM, Littman BH. Gene expression (collagenase, tissue inhibitor of metalloproteinases, complement, and HLA-DR) in rheumatoid arthritis and osteoarthritis synovium. *Arthritis Rheum* 1991; **34**: 1094–1105.
51. Gravallese EM, Darling JM, Ladd AL, Katz JN, Glimcher LH. *In situ* hybridization studies of stromelysin and collagenase messenger RNA expression in rheumatoid synovium. *Arthritis Rheum* 1991; **34**: 1076–1084.
52. McCachren SS. Expression of metalloproteinases and metalloproteinase inhibitor in human arthritic synovium. *Arthritis Rheum* 1991; **34**: 1085–1093.
53. Dayer JM, Ricard-Blum S, Kaufmann MT, Herbage D. Type IX collagen is a potent inducer of PGE2 and interleukin 1 production by human macrophages. *FEBS Lett* 1986; **198**: 208–212.
54. Dayer JM, Trentham DE, Krane SM. Collagens act as ligands to stimulate monocytes to produce mononuclear cell factor (MCF) and protaglandins (PGE2). *Collagen Rel Res* 1982; **2**: 523–540.
55. Morgan K, Clague RB, Collins L, Ayad S, Phinn SD, Holt PJ. Incidence of antibodies to native and denatured cartilage collagens (types II, IX and XI) and to type I collagen in rheumatoid arthritis. *Ann Rheum Dis* 1987; **46**: 902–907.
56. Boissier M-C, Chiocchia G, Texier B, Fournier C. Pattern of humoral reactivity to type II collagen in rheumatoid arthritis. *Clin Exp Immunol* 1989; **78**: 177–183.
57. Cooke TD, Hurd ER, Jasin HE, Bienenstock J, Ziff M. Identification of immunoglobulins and complement in rheumatoid articular collagenous tissues. *Arthritis Rheum* 1975; **18**: 541–551.
58. Kimball ES Substance P, cytokines, and arthritis. *Ann NY Acad Sci* 1990; **594**: 293–308.
59. Lotz M, Vaughan JH, Carson DA. Effect of neuropeptides on production of inflammatory cytokines by human monocytes. *Science* 1988; **241**: 1218–1221.
60. Lotz M, Carson DA, Vaughan JH. Substance P activation of rheumatoid synoviocytes: neural pathway in pathogenesis of arthritis. *Science* 1987; **235**: 893–895.
61. Guerne C, Carson DA, Lotz M. IL-6 production by human articular chondrocytes. Modulation of its synthesis by cytokines, growth factors, and hormones *in vitro*. *J Immunol* 1990; **144**: 499–505.
62. Martel-Pelletier J, Cloutier JM, Pelletier JP. Neutral proteases in human osteoarthritic synovium. *Arthritis Rheum* 1986; **29**: 1112–1121.
63. Wood DD, Ihrie EJ, Dinarello CA, *et al.* Isolation of interleukin 1 like factor from human joint effusions. *Arthritis Rheum* 1983; **26**: 975–983.
64. Dayer JM, Breard J, Chess L, Krane SM. Participation of monocyte–macrophages and lymphocytes in the production of a factor that stimulates collagenase and prostaglandin release by rheumatoid synovial cells. *J Clin Invest* 1979; **64**: 1386–1392.
65. Dayer JM, de Rochemonteix B, Burrus B, Demczuk S, Dinarello CA. Human recombinant interleukin 1 stimulates collagenase and prostaglandin E2 production by human synovial cells. *J Clin Invest* 1986; **77**: 645–648.
66. McCroskery PA, Arai S, Amento EP, Krane SM. Stimulation of procollagenase synthesis in human rheumatoid synovial fibroblasts by mononuclear cell factor/ interleukin 1. *FEBS Lett* 1985; **191**: 7–12.
67. Goldring MB, Krane SM. Modulation by recombinant interleukin 1 of synthesis of types I and III collagens and associated procollagen mRNA levels in culture human cells. *J Biol Chem* 1987; **262**: 16724–16729.
68. Krane SM, Dayer JM, Simon LS, Byrne MS. Mononuclear cell-conditioned medium containing mononuclear cell factor (MCF), homologous with interleukin 1, stimulates collagen and fibronectin synthesis by adherent rheumatoid synovial cells: effects of prostaglandin $E_2$ and indomethacin. *Coll Rel Res* 1985; **5**: 99–117.
69. Kohase M, Henriksen-DeStefano D, May LT, Vilcek J. Induction of B2-interferon by tumor necrosis factor. A homeostatic mechanism in the control of cell proliferation. *Cell* 1986; **45**: 659–666.
70. Lotz M, Guerne PA. Interleukin-6 induces the synthesis of tissue inhibitor of metalloproteinases-1/erythroid potentiating activity (TIMP1/EPA). *J Biol Chem* 1991; **266**: 2017–2020.
71. Springer TA. Adhesion receptors of the immune system. *Nature* 1990; **346**: 425–434.
72. Tessier P, Audette M, Cattaruzzi P, McColl SR. Up-regulation by tumor necrosis factor of intercellular adhesion molecule 1 expression and function in synovial fibroblasts and its inhibition by glucocorticoids. *Arthritis Rheum* 1993; **11**: 1528–1539.
73. Konttinen YT, Gronblad M, Bergroth V, *et al.* Presence of platelet glycoproteins Ib and IIb-IIIa in inflammatory and noninflammatory synovium. *J Rheumatol* 1989; **16**: 578–584.
74. Schooltink H, Stoyan T, Lenz D, *et al.* Structural and functional studies on the human hepatic IL-6-receptor: molecular cloning and overexpression in HepG2 cells. *Biochem J* 1991; **277**: 659–664.
75. Oppenheim JJ, Zacharie CO, Mukaida N, Matsushima K. Properties of the novel proinflammatory supergene 'intercrine' cytokine family. *Ann Rev Immunol* 1991; **9**: 617–648.
76. Yoshimura T, Matsushima K, Oppenheim JJ, Leonard EJ. Neutrophil chemotactic factor produced by lipopolysaccharide (LPS)-stimulated human blood mononuclear leukocytes: partial characterization and separation from interleukin 1 (IL-1). *J Immunol* 1987; **139**: 788–793.
77. Strieter RM, Chensue SW, Bashna MA, *et al.* Human alveolar macrophage gene expression of interleukin-8 by tumor necrosis factor-alpha, lipopolysaccharide, and interleukin-1 beta. *Am J Respir Cell Mol Biol* 1990; **2**: 321–326.
78. Elner VM, Strieter RM, Elner SG, Baggiolini M, Lindley I, Kunkel SL. Neutrophil chemotactic factor (IL-8) gene expression by cytokine-treated retinal pigment epithelial cells. *Am J Pathol* 1990; **136**: 745–750.
79. Strieter RM, Phan SH, Showell HJ, *et al.* Monokine-induced neutrophil chemotactic factor gene expression in human fibroblasts. *J Biol Chem* 1989; **264**: 10621–10626.
80. Watson ML, Lewis GPW. Neutrophil stimulation by recombinant cytokines and a factor produced by IL-1 treated human synovial cell cultures. *Immunology* 1988; **65**: 567–572.
81. Brennan FM, Zachariae COC, Chantry D, et al. Detection of interleukin-8 biologic activity in synovial fluids from patients with rheumatoid arthritis and production of interleukin-8 mRNA by isolated synovial cells. *Eur J Rheum* 1990; **20**: 2141–2144.
82. Rathanaswami P, Hachina M, Sadick M, Schall TJ, McColl SR. Expression of the cytokine RANTES in human rheumatoid synovial fibroblasts. Differential regulation of RANTES in human rheumatoid synovial fibroblasts. *J Biol Chem* 1993; **268**: 5834–5839.
83. Koch AE, Kunkel SL, Burrows JC, *et al.* Synovial tissue macrophage as a source of the chemotactic cytokine IL-8. *J Immunol* 1991; **147**: 2187–2195.
84. Seitz M, Dewald B, Gerber N, Baggiolini M. Enhanced production of neutrophil-activating peptide-interleukin-8 in rheumatoid arthritis. *J Clin Invest* 1991; **87**: 463–469.
85. Villiger PM, Terkeltaub R, Lotz M. Production of monocyte chemoattractant protein-1 by inflamed synovial tissue and cultured synoviocytes. *J Immunol* 1992; **149**: 722–727.
86. Goldring MB, Birkhead J, Sandell LJ, Kimura T, Krane SM. Interleukin-1 suppresses expression of cartilage-specific types II and IX collagens and increases types I and III collagens in human chondrocytes. *J Clin Invest* 1988; **82**: 2026–2037.
87. Tyler JA. Articular cartilage cultured with calabolin (pig interleukin-1) synthesized a decreased number of normal proteoglycan molecules. *Biochem J* 1985; **227**: 869–878.
88. Nietfeld JJ, Wilbrink B, Helle M, *et al.* Interleukin-1 induced interleukin-6 is required for the inhibition of proteoglycan synthesis by interleukin-1 in human articular cartilage. *Arthritis Rheum* 1990; **33**: 1695–1701.
89. Bender S, Haubeck HD, Van de Leur E, *et al.* Interleukin-l beta induces synthesis and secretion of interleukin-6 in human chondrocytes. *FEBS Lett* 1990; **263**: 321–324.
90. Yaron I, Meyer FA, Dayer JM, Yaron M. Human recombinant interleukin-1 beta stimulates glycosaminoglycan production in human synovial fibroblast cultures. *Arthritis Rheum* 1987; **30**: 424–430.
91. Heino J, Kahari VM, Mauviel A, Krusius T. Human recombinant interleukin-1 regulates cellular mRNA levels of dermatan sulphate proteoglycan core protein. *Biochem J* 1988; **252**: 309–312.
92. Campbell IK, Piccoli DS, Hamilton JA. Stimulation of human chondrocyte prostaglandin $E_2$ production by recombinant human interleukin-1 and tumour necrosis factor. *Biochim Biophys Acta* 1990; **1051**: 310–318.
93. Pratta MA, Di Meo TM, Ruhl DM, *et al.* Effect of interleukin-1 and tumour necrosis factor-alpha on cartilage proteoglycan metabolism in vitro. *Agents Actions* 1989; **27**: 250–253.
94. Saklatvala J. Tumour necrosis factor alpha stimulates resorption and inhibits synthesis of proteoglycan in cartilage. *Nature* 1986; **322**: 547–549.
95. Lefebvre V, Peeters-Joris C, Vaes G. Modulation of interleukin 1 and tumor necrosis factor alpha of production of collagenase, tissue inhibitor of metalloproteinase and collagen types in differentiated and dedifferentiated articular chondrocytes. *Biochim Biophys Acta* 1990; **1052**: 366–378.
96. Kandel RA, Petelycky M, Dinarello CA, *et al.* Comparison of the effect of interleukin 6 and interleukin 1 on collagenase and proteoglycan production by chondrocytes. *J Rheumatol* 1990; **17**: 953–957.
97. Sato T, Ito A, Mori Y. Interleukin 6 enhances the production of tissue inhibitor of metalloproteinase (TIMP) but not that of matrix metalloproteinases by human fibroblasts. *Biochem Biophys Res Commun* 1990; **170**: 824–829.
98. Guerne PA, Vaughan JH, Carson DA, et al. Interleukin 6 (IL-6) and joint tissues. *Ann NY Acad Sci* 1989; **557**: 558–561.
99. Martel-Pelletier J, Zafarullah M, Kodama S, *et al. In vitro* effects of IL-1 on the synthesis of metalloprotease, TIMP, plasminogen activators and inhibitors in human articular cartilage. *J Rheumatol* 1991; **18**: 80–84.
100. Bunning RAD, Crawford A, Richardson HJ, *et al.* Interleukin-1 preferentially stimulates the production of tissue-type plasminogen activator by human articular chondrocytes. *Biochim Biophys Acta* 1990; **1051**: 310–318.
101. Campbell IK, Last K, Novak U, *et al.* Recombinant human interleukin-1 inhibits plasminogen activator inhibitor-1 (PAI-1) production by human articular cartilage and chondrocytes. *Biochem Biophys Res Commun* 1991; **174**: 251–257.
102. Mochan E, Keler T. Plasmin degradation of cartilage proteoglycan. *Biochim Biophys Acta* 1984; **800**: 312–315.
103. Dinarello CA. Anti-cytokine strategies. *Eur Cytokine Network* 1992; **3**: 7–17.
104. Cannon JG, Nerad JL, Poutsiaka DD, Dinarello CA. Measuring circulating cytokines. *J Appl Physiol* 1993; **75**: 1897–1902.
105. Ganapathi MK, Schultz D, Màckiewicz A, *et al.* Heterogenous nature of the acute phase response. Differential regulation of human serum amyloid A, C-reactive protein, and other acute phase proteins by cytokines in Hep 3B cells. *J Immunol* 1988; **141**: 564–569.
106. Otterness IG. The clinical value of C-reactive protein (CRP) measurement in the management of rheumatoid arthritis. *Seminars Arthritis Rheum* 1994; (in press).
107. Rokita H, Loose LD, Bartle LM, Sipe JD. Synergism of interleukin 1 and interleukin 6 induces serum amyloid A (SAA) production while depressing fibrinogen: a quantitative analysis. *J Rheum* 1994; **21**: 400–405.
108. McConkey B, Crockson RA, Crockson AP. The assessment of rheumatoid arthritis: a study based on measurements of serum acute phase reactants. *Quart J Med* 1972; **41**: 115–125.
109. McConkey B, Crockson RA, Crockson AP, *et al.* The effects of some anti-inflammatory drugs on the acute-phase proteins in rheumatoid arthritis. *Quart J Med* 1973; **42**: 785–791.
110. Larsen A. The relation of radiographic changes to serum acute-phase proteins and rheumatoid factor in 200 patients with rheumatoid arthritis. *Scand J Rheum* 1988; **17**: 123–129.
111. Chambers RE, Macfarlane DG, Whicher JT, Dieppe PA. Serum amyloid-A protein concentration in rheumatoid arthritis and its role in monitoring disease activity. *Ann Rheum Diseases* 1983; **42**: 665–667.
112. Sukenik S, Henkin J, Zimlichman S, *et al.* Serum and synovial fluid levels of serum amyloid A protein and C-reactive protein in inflammatory and noninflammatory arthritis. *J Rheum* 1988; **15**: 942–945.
113. Loose LD, de Oliveira RM, J Sipe, Franzblau C, Shanahan WR. A possible systemic component of osteoarthritis (OA): elevated concentrations (by ELISA) of C-reactive protein (CRP) in serum of OA patients and modulation by tenidap. *Arthritis Rheum* 1993; **36**: S166.
114. Fontana A, Hengartner H, Wever E, Fehr K, Grob PJ, Cohen G. Interleukin-1 activity in the synovial fluid of patients with rheumatoid arthritis. *Rheumatol Int* 1982; **2**: 49–53.
115. Macnaul KL, Hutchinson NI, Parsons JN, Bayne EK, Tocci MJ. Analysis of IL1 and TNF alpha gene expression in human rheumatoid synoviocytes and normal monocytes by *in situ* hybridization. *J Immunol* 1990; **145**: 4154–4166.
116. Duff GW, Dickens E, Wood N, *et al.* Immunoassay, bioassay, and *in situ* hybridization of monokines in human arthritis. *Progr Leucocyte Biol* 1988; **8**: 387–392.
117. Buchan G, Barrett K, Turner M, Chantry D, Maini RN, Feldmann M. Interleukin 1 and tumor necrosis factor mRNA expression in rheumatoid arthritis: prolonged production of IL-1alpha. *Clin Exp Immunol* 1988; **73**: 449–455.
118. Chu CQ, Field M, Feldmann M, Maini RN. Localization of tumor necrosis factor alpha in synovial tissues and at the cartilage-pannus junction in patients with rheumatoid arthritis. *Arthritis Rheum* 1991; **34**: 1125–1132.
119. Field M, Chu C, Feldman M, Maini RN. Interleukin-6 localization in the synovial membrane in rheumatoid arthritis. *Rheumatol Int* 1991; **11**: 45–50.
120. Firestein GS, Alvaro-Garcia JM, Make R. Quantitative analysis of cytokine gene expression in rheumatoid arthritis. *J Immunol* 1990; **144**: 3347–3353.
121. Tan PL, Farmiloe S, Yeoman S, Watson JD. Expression of the interleukin 6 gene in rheumatoid synovial fibroblasts. *J Rheumatol* 1990; **17**: 1608–1612.
122. Wood NC, Symons JA, Dickens E, Duff GW. *In situ* hybridization of IL-6 in rheumatoid arthritis. *Clin Exp Immunol* 1992; **87**: 183–189.
123. Hirano T, Matsuda T, Turner M, *et al.* Excessive production of interleukin 6/B cell stimulatory factor-2 in rheumatoid arthritis. *Eur J Immunol* 1988; **18**: 1797–1801.
124. Xu W, Firestein GS, Taetle R, Kaushansky K, Zvaifler NJ. Cytokines in chronic inflammatory arthritis, II: Granulocyte-macrophage colony-stimulating factor in rheumatoid synovial effusions. *J Clin Invest* 1989; **83**: 876–882.
125. Brennan FM, Chantry D, Turner M, Foxwell B, Maini RN, Feldman M. Detection of transforming growth factor-beta in rheumatoid arthritis synovial tissue: lack of effect on spontaneous cytokine production in joint cell cultures. *Clin Exp Immunol* 1990; **81**: 278–285.
126. Arend WP, Dayer J-M. Cytokines and cytokine inhibitors or antagonists in rheumatoid arthritis. *Arthritis Rheum* 1990; **33**: 305–315.
127. Feldmann M, Brennan FM, Chantry D, *et al.* Cytokine production in the rheumatoid joint: implications for treatment. *Ann Rheum Dis* 1990; **49**: 480–486.
128. Otterness IG, van de Loo FAJ, Bliven ML. Cytokines in models of arthritis. In: Henderson B, Pettipher ER, Edwards J, eds. *Mechanisms and Models of Rheumatoid Arthritis.* 1994.
129. Carswell EA, Old LJ, Kasse R, Green S, Fiore N, Williamson B. An endotoxin-induced serum factor that causes necrosis of tumors. *Proc Natl Acad Sci USA* 1975; **72**: 3666–3670.
130. Aggarwal BB. Tumor necrosis factors—TNF alpha and TNF beta: their structure and pleiotropic biological effects. *Drugs of the Future* 1987; **12**: 891–898.
131. Beutler B, Cerami A. Tumor necrosis, cachexia, shock and chronic inflammation. *Ann Rev Biochem* 1988; **57**: 505–518.
132. Vilcek J, Lee TH. Tumor necrosis factor. New insights into the molecular mechanisms of its multiple actions. *J Biol Chem* 1991; **266**: 7313–7316.
133. Bonta IL, Ben-Efraim S, Mozes T, Fieren MWJA. Tumor necrosis factor in inflammation: relation to other mediators and to macrophage antitumour defence. *Pharmacological Research* 1991; **24**: 115–130.
134. Keffer J, Probert L, Cazlaris H, *et al.* Transgenic mice expressing human tumour necrosis factor: a predictive genetic model of arthritis. *EMBO J* 1991; **10**: 4025–4031.
135. Henderson B, Pettipher E. Arthritogenic actions of recombinant IL-1 and tumour necrosis factor alpha in the rabbit: evidence for synergistic interactions between cytokines *in vivo*. *Clin Exp Immunol*; 1989; **75**: 306–310.
136. O'Byrne EM, Blancuzzi V, Wilson DE, Wong M, Jeng AY. Elevated substance P and accelerated cartilage degradation in rabbit knees injected with interleukin-1 and tumor necrosis factor. *Arthritis Rheum* 1990; **33**: 1023–1028.
137. Loetscher H, Pan Y-C, Lahm H-W, *et al.* Molecular cloning and expression of human 55 kDa tumor necrosis factor receptor. *Cell* 1990; **61**: 351–359.
138. Smith CA, Davis T, Anderson D, *et al.* A receptor for tumor necrosis factor defines an unusual family of cellular and viral proteins. *Science* 1990; **248**: 1019–1023.
139. Kronke M, Schutze S, Scheurich P, Pfizenmaier K. TNF signal transduction and TNF-responsive genes. In: *Tumor Necrosis Factor: Structure, Function and Mechanism of Action.* 1991; 189-216.
140. Deluran BW, Chu C-Q, Field M, *et al.* Localization of tumor necrosis factor receptors in the synovial tissue and cartilage-pannus junction in patients with rheumatoid arthritis. Implications for local actions of tumor necrosis factor alpha. *Arthritis Rheum* 1992; **35**: 1170–1178.
141. Han J, Brown T, Beutler B. Endotoxin-responsive sequences control cachectin/tumor necrosis factor biosynthesis at the translational level. *J Exp Med* 1990; **171**: 465.
142. Perez C, Albert I, DeFay K, Zachariaded N, Gooding L, Kriegler M. A non-secretable cell surface mutant of tumor necrosis factor (TNF) kills by cell-to-cell contact. *Cell* 1990; **63**: 251–258.
143. Sioud M, Drlica K. Prevention of human immunodeficiency virus type I integrase expression in *Escherichia coli* by a ribozyme. *Proc Natl Acad Sci* 1991; **88**: 7303–7307.
144. Sullenger BA, Cech TR. Tethering ribozymes to a retroviral packaging signal for destruction of viral RNA. *Science* 1993; **262**: 1566–1569.
145. Sioud M, Natvig JB, Forre O. A preformed ribozyme destroys cytokine mRNA in human cells. *Arthritis Rheum* 1993; **36**: S267.
146. Alzani R, Corti A, Grazioli L, Cozzi E, Ghezzi P, Marcucci F. Suramin induces deoligomerization of human tumor necrosis factor alpha. *J Biol Chem* 1993; **268**: 12526–12529.
147. Grazioli L, Alzani R, Ciomei M, *et al.* Suramin. *Int J Immunopharmacol* 1992; **14**: 637–642.
148. Elliott MJ, Maini RN, Feldmann M, *et al.* Treatment of rheumatoid arthritis with chimeric monoclonal antibodies to tumor necrosis factor alpha. *Arthritis Rheum* 1993; **36**: 1681–1690.
149. Engelmann H, Aderka D, Rubinstein M, Rotman D, Wallach D. A tumor necrosis factor-binding protein purified to homogeneity from human urine protects cells from tumor necrosis factor toxicity. *J Biol Chem* 1989; **264**: 11974–11980.
150. Engelmann H, Holtmann H, Brakebusch C, *et al.* Antibodies to a soluble form of a tumor necrosis factor (TNF) receptor have TNF-like activity. *J Biol Chem* 1990; **265**:14497-14504.
151. Engelmann H, Novick D, Wallach D. Two tumor necrosis factor-binding proteins purified from human urine. Evidence for immunological cross-reactivity with cell surface tumor necrosis factor receptors. *J Biol Chem* 1990; **265**: 1531–1536.
152. Seckinger P, Zhang JH, Hauptmann B, Dayer JM. Characterization of a TNF-alpha inhibitor. Evidence of immunological cross-reactivity with the TNF receptor. *Proc Natl Acad Sci* 1990; **87**: 5188–5192.
153. Cope AP, Aderka D, Doherty M, *et al.* Increased levels of soluble tumor necrosis factor receptors in the sera and synovial fluid of patients with rheumatic diseases. *Arthritis Rheum* 1990 **35**; 1160–1169.
154. Piguet PF, Grau GE, Vesin C, Loetscher H, Gentz R, Lesslauer W. Evolution of collagen arthritis in mice is arrested by treatment with antitumor necrosis factor (TNF) antibody or a recombinant soluble TNF receptor. *Immunology* 1992; **77**: 510–514.
155. Williams RO, Feldmann M, Maini RN. Anti-tumor necrosis factor ameliorates joint disease in murine collagen-induced arthritis. *Proc Natl Acad Sci* 1991; **89**: 9784–9788.
156. Pettipher ER, Higgs GA, Henderson B. Interleukin 1 induces leukocyte infiltration and cartilage proteoglycan degradation in the synovial joint. *Proc Natl Acad Sci* 1986; **83**: 8749–8753.
157. Stimpson SA, Dalldorf FG, Otterness IG, Schwab JH. Exacerbation of arthritis by IL-1 in rat joints previously injured by peptidoglycan–polysaccharide. *J Immunol* 1988; **140**: 2964–2969.
158. Hom JT, Bendele AM, Carlson DG. *In vivo* administration with IL-1 accelerates the development of collagen-induced arthritis in mice. *J Immunol* 1988; **141**: 834–841.
159. Dinarello CA. Interleukin-1 and interleukin-1 antagonism. *Blood* 1991; **77**: 1627–1652.
160. Sipe JD. The molecular biology of interleukin 1 and the acute phase response. In: Stollerman GH, ed. *Adv Intern Med.* Year Book Medical Publishers, 1989; 1–20.
161. Fenton MJ. Transcriptional factors that regulate human IL-1/hemopoietin 1 gene expression. *Hematopoesis* 1990; **120**: 67–82.
162. Fenton MJ. Transcriptional and post-transcriptional regulation of interleukin 1 gene expression. *Int J Immunopharm* 1992; **14**: 401–411.
163. Beuscher HU, Rausch U-P, Otterness IG, Rollinghoff M. Transition from interleukin 1beta (IL-1beta) to IL-1alpha production during maturation of inflammatory macrophages *in vivo. J Exp Med* 1992; **175**: 1793–1797.
164. Holtmann H, Wallach D. Down regulation of the receptors for tumor necrosis factor by interleukin-1 and -4 beta-phorbol-12-myristate-13-acetate. *J Immunol* 1987; **139**: 1161–1167.
165. Wallach D, Holtmann H, Aderka D, *et al.* Mechanisms which take part in regulation of the response to tumor necrosis factor. *Lymphokine Res* 1989; **8**: 359–363.
166. Collata F, Re F, Bertini R, *et al.* Interleukin type II receptor: a decoy target for IL-1 that is regulated by interleukin 4. *Science* 1993; **261**: 472–475.
167. Symons JA, Eastgate JA, Duff GW. Purification and characterization of a novel soluble receptor for interleukin-1. *J Exp Med* 1991; **174**: 1251–1254.
168. Fanslow WC, Sims JE, Sassenfeld H, *et al.* Regulation of alloreactivity *in vivo* by a soluble form of the interleukin-1 receptor. *Science* 1990; **248**: 739–742.
169. Dinarello CA, Thompson RC. Blocking IL-1: interleukin 1 receptor antagonist *in vivo* and *in vitro. Immunol Today* 1991; **12**: 404–410.
170. Dubois CM, Ruscetti FW, Palaszynski EW, Falk LA. Transforming growth factor beta is a potent inhibitor of interleukin-1 (IL-1) receptor expression: proposed mechanism of inhibition of IL-1 action. *J Exp Med* 1990; **172**: 737–742.
171. van de Loo FAJ, Arntz OJ, Otterness IG, van den Berg WB. Protection against cartilage proteoglycan synthesis inhibition by antiinterleukin 1 antibodies in experimental arthritis. *J Rheumatol* 1992; **19**: 348–356.
172. Lebsack ME, Paul CC, Bloedow DC, *et al.* Subcutaneous IL-1 receptor antagonist in patients with rheumatoid arthritis. *Arthritis Rheum* 1991; **34**: S45.
173. Prospects for antisense nucleic acid therapy of cancer and AIDS. In: Wickstrom D, ed. Wiley-Liss. New York: Wiley-Liss, 1991; 1–267.
174. Maier JAM, Voulalas P, Roeder D, Maciag T. Extension of the life span of human endothelial cells by an interleukin-1 alpha antisense oligomer. *Science* 1990; **249**: 1570–1574.
175. Budker VG, Knorre DG, Vlassov VV. Cell membranes as barriers for antisense oligonucleotides. *Antisense Res Dev* 1992; **2**: 177–184.
176. Bandara G, Mueller GM, Galea-Lauri JT, *et al.* Intraarticular expression of biologically active interleukin 1-receptor-antagonist protein by *ex vivo* gene transfer. *Proc Natl Acad Sci USA* 1993; **90**: 10764–10768.
177. Le J, Vilcek J. Interleukin 6: a multifunctional cytokine regulating immune reactions and the acute phase protein response. *Lab Invest* 1989; **61**: 588–602.
178. Heinrich PC, Castell JV, Andus T. Interleukin-6 and the acute phase response. *Biochem J* 1990; **265**: 621–636.
179. Rose-John S, Heinrich PC. Interleukin-6 receptor. In: Mackiewicz A, Kushner I, Baumann H, eds. *Acute Phase Proteins—Molecular Biology, Biochemistry, Clinical Applications* 1993; 343–361.
180. Gearing DP, Comeau MR, Friend DJ, *et al.* The IL-6 signal transducer, gp130: an oncostatin M receptor and affinity converter for the LIF receptor. *Science* 1992; **255**: 1434–1436.
181. Novick D, Engelmann H, Wallach D, Rubinstein M. Soluble cytokine receptors are present in normal human urine. *J Exp Med* 1989; **170**: 1409–1414.
182. Novick D, Engelmann H, Wallach D, Leitner O, Revel M, Rubinstein M. Purification of soluble cytokine receptors from normal human urine by ligand affinity and immunoaffinity chromatography. *J Chromat* 1990; **510**: 331–337.
183. Bonta IL, Parnham MJ, Vincent JE, Bragt PC. Anti-rheumatic drugs: present deadlock and new vistas. *Progress in Medicinal Chemistry* 1980; **17**: 186–273.
184. Goodwin JS, Ceuppens JL. Effect of nonsteroidal antiinflammatory drugs on immune function. *Semin Arthritis Rheum* 1983; **13**: S134–S143.
185. Gordon D, Lewis GP. Effects of piroxicam on mononuclear cells. Comparison with other antiarthritic drugs. *Inflammation* 1984; **8**: S87–S102.
186. Cesario TC, Yousefi S, Carandang G. The regulation of interferon production by aspirin and other inhibitors of the cyclooxygenase pathway and agents influencing calcium channel flux. *Bull NY Acad Med* 1989; **65**: 26–35.
187. Rappaport RS, Dodge GR. Prostaglandin E inhibits the production of human interleukin 2. *J Exp Med* 1982; **155**: 943–948.
188. Snijdewint FGM, Kalinski P, Wierenga EA, Bos JD, Kapsenberg ML. Prostaglandin $E_2$ differentially modulates cytokine secretion profiles of human T helper lymphocytes. *J Immunol* 1993; **150**: 5321–5329.
189. Haynes DR, Whitehouse MW, Vernon-Roberts B. The prostaglandin $E_1$ analogue, misoprostol, regulates inflammatory cytokines and immune functions *in vitro* like the natural prostaglandins $E_1$, $E_2$, and $E_3$. *Immunology* 1992; **76**: 251–257.
190. Mochan E, Uhl J, Newton R. Evidence that interleukin-1 induction of synovial cell plasminogen activator is mediated by prostaglandin E2 and cyclic AMP. *Arthritis Rheum* 1986; **29**: 1078-1084.
191. Meade EA, Smith WL, DeWitt DL. Differential inhibition of prostaglandin endoperoxide synthase (cyclooxygenase) isozymes by aspirin and other nonsteroidal antiinflammatory drugs. *J Biol Chem* 1993; **344**: 4610–4614.
192. Garcia-Leme J, Farsky SP. Hormonal control of inflammatory responses. *Mediators of Inflammation* 1993; **2**: 181–198.
193. Nishida T, Nakai S, Kawakami T, Nishino N, Nakai S, Hirai Y. The transcription of the interleukin 1 beta gene is induced with PMA and inhibited with dexamethasone in U937 cells. *Biochem Biophys Res Commun* 1988; **156**: 269–274.
194. Knudsen PJ, Dinarello CA, Strom TB. Glucocorticoids inhibit transcriptional and post-transcriptional expression of interleukin 1 in U937 cells. *J Immunol* 1987; **139**: 4129–4134.
195. Lee SW, Tsou A, Chan H, *et al.* Glucocorticoids selectively inhibit the transcription of the interleukin 1 beta gene and decrease the stability of interleukin 1 beta mRNA. *Proc Natl Acad Sci* 1988; **85**: 1204–1208.
196. Lee JC, Griswold DE, Votta B, Hanna N. Inhibition of monocyte IL-1 production by the anti-inflammatory compound SK&F 86002. *Int J Immunopharmacol* 1988; **10**: 835–843.
197. Arya SK, Wong-Staal F, Gallo RC. Dexamethasone-mediated inhibition of human T cell growth factor and gamma-interferon messenger RNA. *J Immunol* 1984; **133**: 273–276.
198. Culpepper JA, Lee F. Regulation of IL-3 expression by glucocorticoids in cloned murine T lymphocytes. *J Immunol* 1985; **135**: 3191–3197.
199. Tanabe O, Akira S, Kamiya G, Wong GG, Hirano T, Kishimoto T. Genomic structure of the murine IL-6 gene: high degree conservation of potential regulatory sequences between mouse and human. *J Immunol* 1988; **141**: 3875–3881.
200. Culpepper JA, Lee F. Glucocorticoid regulation of lymphokine production by murine T lymphocytes. *Lymphokine* 1987; **13**: 275–281.
201. Waage A, Bakke O. Glucocorticoids suppress the production of tumor necrosis factor alpha by lipopolysaccharide stimulated human monocytes. *Immunology* 1988; **63**: 299–302.
202. Arend WP, Massoni RJ. Characteristics of bacterial lipopolysaccharide induction of interleukin-1 secretion by lipopolysaccharides. *Clin Exp Immunol* 1986; **64**: 656–664.
203. Kern JA, Lamb RJ, Reed JC, Daniele RP, Nowell PC. Dexamethasone inhibition of interleukin-1 beta production by human monocytes: posttranscriptional mechanisms. *J Clin Invest* 1988; **81**: 237–294.
204. Hurme M, Siljander P, Anttila H. Regulation of interleukin-1 beta production by glucocorticoids in human monocytes: the mechanism of action depends on the activation signal. *Biochem Biophys Res Commun* 1991; **180**: 1383–1389.
205. Bogdan C, Vodovotz Y, Nathan C. Macrophage deactivation by interleukin-10. *J Exp Med* 1991; **174**: 1549–1555.
206. de Waal Malefyt R, Abrams J, Bennert B, Figdor G, de Vries J. Interleukin-10 (IL-10) inhibits cytokine synthesis by human monocytes: an autoregulatory role of IL-10 produced by monocytes. *J Exp Med* 1991; **174**: 1209.
207. Kyle V, Coughlan RJ, Tighe H, Waldmann H, Kazleman BL. Beneficial effect of monoclonal antibody to interleukin 2 receptors on activated T cells on rheumatoid arthritis. *Ann Rheum Dis* 1989; **48**: 428–429.
208. Trentham DE, Dynesius-Trentham RA, Orav EJ, *et al.* Effects of oral administration of type II collagen on rheumatoid arthritis. *Science* 1993; **261**: 1727–1730.
209. Seow WK, Nakamura K, Sugimura Y, *et al.* Inhibitory effects of bis-benzylisoquinolines on synthesis of the inflammatory cytokines interleukin-1 and tumour necrosis factor-alpha. *Mediators of Inflammation* 1993; **2**: 199–203.
210. Tao X, Cai J, Lipsky PE. Biological effects of the ethyl acetate extract of *Tripterygium wilfordii* Hook F. *Arthritis Rheum* 1993; **36**: S266.
211. Gu W-Z, Chen R, Brandwein S, McAlpine J, Burres N, Rubin P. Isolation, purification, and characterization of novel immunosuppressive compounds from *Tripterygium. Arthritis Rheum* 1993; **36**: S228.
212. Ingber D, Fujita T, Kishimoto S, *et al.* Synthetic analogues of fumagilin that inhibit angiogenesis and suppress tumour growth. *Nature* 1990; **348**: 555–557.
213. Peacock DJ, Banquerigo ML, Brahn E. Angiogenesis inhibition suppresses collagen arthritis. *J Exp Med* 1992; **175**: 1135–1138.
214. Yu LPJ, Smith GNJ, Brandt KD, Myers SL, O'Connor BL, Brandt DA. Reduction of the severity of canine osteoarthritis by prophylactic treatment with oral doxycycline. *Arthritis Rheum*, 1992; **35**: 1150–1159.
215. Greenwald RA, Moak SA, Ramamurthy NS, Golub LM. Tetracyclines suppress matrix metalloproteinase activity in adjuvant arthritis and in combination with flurbiprofen, ameliorate bone damage. *J Rheumatol* 1992; **19**: 927–938.
216. Otterness IG, Bliven ML, Carreras I, Sipe JD. *In vivo* roles of IL-1 alpha and IL-1 beta. *Cytokine* 1991; **3**: 513.
217. Sipe JD, Bartle LM, Loose LD. Modification of proinflammatory cytokine production by the antirheumatic agents tenidap and naproxen. A possible correlate with clinical acute phase response. *J Immunol* 1992; **148**: 480–484.
218. Dougados M, Combe B, Beveridge T, *et al.* IX207-887 in rheumatoid arthritis. A double-blind placebo-controlled study. *Arthritis Rheum* 1992; **35**: 999–1006.
219. Davis JS, Loose LD, Borger AP. Clinical efficacy of CP-66,248 [5-chloro-2,3-

dihydro-2-oxo-3-(2-theinylcarbonyl)-indole-1-carboxamide] in osteoarthritis (OA). *Arthritis Rheum* 1988; **31**: S72.
220. Katz PB, Borger AP, Loose LD. Evaluation of CP-66,248 [5-chloro-2,3-dihydro-2-oxo-3-(2-theinylcarbonyl)-indole-1-carboxamide] in rheumatoid arthritis (RA). *Arthritis Rheum* 1988; **31**: S52.
221. Delmas PD. Clinical use of biochemical markers of bone remodeling in osteoporosis. *Bone* 1992; **13**: S17–S21.
222. Pelletier J-P, McCollum R, DiBattista J, Loose LD, Cloutier J-M, Martel-Pelletier J. Regulation of human normal and osteoarthritic chondrocyte interleukin-1 receptor by antirheumatic drugs. *Arthritis Rheum* 1993; **36**: 1517-1527.
223. Loose LD, Sipe JD, Kirby DS, *et al.* Reduction of acute phase proteins with tenidap, a cytokine-modulating antirheumatic drug. *Br J Rheum* 1993; **32**(suppl 3): 19–25.
224. Rainsford KD. Effects of antimalarial drugs on interleukin 1-induced cartilage proteoglycan degradation *in vitro*. *J Pharm Pharmacol* 1986; **38**: 829–833.
225. Chin J, Kostura MJ. Dissociation of IL-1beta synthesis and secretion in human blood monocytes stimulated with bacterial cell wall products. *J Immunol* 1993; **151**: 5574–5585.
226. Larsen CG, Paludan K, Kristensen M, *et al.* ETH 615, a novel inhibitor of interleukin-8 (IL-8) production. *J Invest Dermatol* 1990; **95**: 478–482.

ACKNOWLEDGEMENTS. The authors thank Dr L. D. Loose for helpful discussions and constructive criticism and Ms L. Tran and Ms M. Soohoo for help with literature searching and preparation of the manuscript.

**Received 19 January 1994; accepted 24 January 1994**

